# Ponce DeLeon Spring

**Published:** 1874-03

**Authors:** 


PONCE DELEON SPRING.—The new Hotel near this famous Spring is occupied by Mrs. H. E. Doyle, a lady peculiarly fitted and gifted to give satisfaction to all who may visit the spring and desire hotel accommodations. Board from $30 to $60 per month. For further information address Mrs. H. E. Doyle, Atlanta, Ga.

